# Bright night sleeping environment induces diabetes and impaired glucose tolerance in non-human primates

**DOI:** 10.3389/fendo.2025.1454592

**Published:** 2025-02-12

**Authors:** Shuxing Wang, Zhenyi Chen, Zihao Liang, Qiang Xu, Jiankai Zhang

**Affiliations:** ^1^ Department of Anatomy, Medical School, Foshan University, Foshan, Guangdong, China; ^2^ BIO-THERA, Guangzhou, China; ^3^ Department of Pharmacy, Qingyuan Hospital of Traditional Chinese Medicine, Qingyuan, Guangdong, China; ^4^ Primate Research Center, Institute of Zoology, Guangdong Academy of Sciences, Guangzhou, China; ^5^ Department of Anatomy, Guangdong Medical University, Dongguan, Guangdong, China

**Keywords:** glucose metabolism, insulin, diabetes, impaired fasting glucose tolerance, light at night, melatonin, monkey

## Abstract

**Background:**

According to the IDF Diabetes Atlas regularly published by International Diabetes Federation, the prevalence of diabetes and impaired glucose tolerance (IGT), diabetes-related mortality and health expenditure are becoming serious eventually at the global, regional and national level. While the data alarm people, the exact cause remains unknown. It is widely accepted that glucose metabolism can be impaired by circadian rhythms disruption and sleep disturbances, both closely linked to exposure to light at night. However, there is little direct experiment on primates to study the precise extent of how serious bright sleeping environment at night impairs glucose metabolism, what the relationship is between nocturnal brightness and the development of diabetes and IGT, any difference between male and female, and whether aging and weight are involved. This study aims to address these questions in monkeys.

**Methods:**

In a reduced daytime bright condition resembling human living rooms, 197 Cynomolgus (130 male, 67 female) were exposed to three distinct light intensities (13, 35, 75Lux) at night for consecutive ten months. Animals were retrospectively divided into four groups according to glucose metabolic status by the end of the experimental session, spontaneous diabetes mellitus (SDM, N=11), light-induced diabetes (LID, N=83), impaired fasting glucose tolerance (IFG, N=36), and normal glucose tolerance (NGT, N=67). Data pertaining to the glucose metabolism such as concentrations of fasting glucose, glycosylated hemoglobin, plasma insulin and C-peptide were collected monthly and analyzed.

**Results:**

1) Bright night exasperated glucose metabolism in individuals with pre-existing diabetes, led to premature death; 2) Stronger white light intensity-dependently induced diabetes and IFG in previous healthy monkeys: the brighter the light, the quicker the metabolism disturbance and IFG developed, and also the higher morbidity of LID and IFG; 3) Exposure to nocturnal light had a synergistic impairing effect on glucose metabolism with aging and weight. 4) Female were more susceptible to night brightness.

**Conclusions:**

Light in sleeping environment exacerbates glucose metabolism in individuals with pre-existing diabetes, leads to IFG and diabetes in healthy primates. Moreover, the harmful effects of bright night on glucose metabolism are synergistic with aging and weight.

## 1 Introduction

Physiological blood glucose is maintained within strict boundaries through mechanisms that increasing glucose output when circulating glucose level drops, and decreasing glucose output and increasing tissue glucose uptake when glucose level rises (1). Any cause that damages this mechanism would disturb the physiological glucose metabolism and lead to pathophysiological conditions such as diabetes mellitus and impaired glucose tolerance (IGT) (2). Among them, diabetes mellitus is a kind of chronic, non-communicable disease that arises when the body is unable to produce sufficient insulin or fails to effectively utilize the available insulin in blood, leading to a common state of hyperglycemia in the affected individual, while IGT is a prediabetic condition but below the diagnostic threshold for diabetes (2). The hyperglycemia, typically indicated by elevated blood glucose levels as reflected by fasting blood glucose (FBG), is the primary and most frequently utilized index of diabetes. Diabetes exists in two primary types: type 1 and type 2 (T2D). Patients with type 1 diabetes experience an insufficiency of insulin production from the pancreatic β-cells (3). In contrast, those with T2D have normal even much higher circulating insulin concentration as compared to healthy individuals, but their bodies are unable to effectively utilize the available insulin, leading to the coexistence of hyperglycemia and hyperinsulinemia simultaneously. Overall, T2D accounts for over 90% of the patients with diabetes and represents a significant global health issue. To make it worse, a significantly larger population falls into the category of IGT, and without appropriate intervention, the IGT condition may gradually progress to T2D (4).

Exposure to a circadian environment of bright day and dark night is essential for our daily health (5, 6), whereas environmental light at night poses harmful effects on living organisms (7–9). Evidences show that glucose metabolism is modulated with circadian rhythms (1, 10–12) and that both sleep and circadian rhythm play crucial roles in maintaining insulin sensitivity (13–15). However, there lacks systematic studies on primates regarding the extent to which glucose metabolism will be impaired by light at night. Additionally, it remains questionable whether light will impair glucose metabolism in synergy with age, body weight, and gender. By now there are multiple diabetes research results from non-primate animals (16). However, non-human primates with long lifespan and large size may serve as the most appropriate analogs for humans. In this study the effect of bright sleeping environment at night on the glucose metabolism will be explored in Cynomolgus, a species known for its glucose metabolism physiological and pathophysiological characteristics that closely resemble those of humans (17).

## 2 Materials and methods

### 2.1 Animals

Totally 197 adult Cynomolgus monkeys (130 male, 67 female, 5-24 years old, mean 13.58, s.d. ± 4.91, see **Supplementary Table S1** for exact number grouped with age; starting body weight 3.4-15.1kg, mean 7.299, s.d. ± 2.178) were enrolled. The experimental protocol was approved by the Institutional Animal Care and Use Committee at the Guangdong Institute of Zoology, Guangdong Academy of Sciences. All monkeys originated from Guangdong Landau Biotech Inc., a company accredited by AAALAC and CNSA for its experimental non-human primate breeding-and-research environment. Immediately prior to the start of the study, baseline data were collect for future statistical analysis. Monkey groups were randomly allocated into rooms based on their prior social relationships. The numbers of monkey in each group was mainly based on the original size of the group before being put into the experimental room. The animal numbers in different rooms were uneven. People who allocated the monkey were blind of the experimental light intensity in the rooms. None of the monkeys’ characteristics, such as age, gender, weight, or glucose metabolism status, were predetermined. Throughout the study period a research team member or veterinary staff would patrol the rooms twice a day to ensure a smooth and safe experimental session. If a monkey was bullied by others or exhibited serious diabetic complication, it would be promptly moved to a normal circadian room with day and night time brightness coming from natural light, treated by a veterinarian, and raised separately in a cage (1.2x1.5x1.5m). However, for statistical purposes, the data already collected from this monkey remained valid.

### 2.2 The living environment

Eleven rooms were utilized, each with dimensions of 15x5x3.5 meters (depth, width, and height respectively). Each room features a top window spanning 3x5 meters, a south-facing back window measuring 0.5x2.5 meters, and a metal net door (5x3.5 meters) that opens to a 2-meter wide corridor on the northern side. In each room there was an ad libitum access food tray (1x1 meter) on the floor, a push-drink distilled water fountain, six level shelves for sitting and playing on the wall, and recreational equipment including a swing, tumbling barrel, log, and balls.

### 2.3 Lighting condition, background noise, and temperature

The experiment was carried out at the north latitude 23°26′. For each experimental room the light during the day came from sunlight filtering through the windows and scattered light. Automatically controlled light tubes on the ceiling of the corridor, which shone through the net door, provided light from 5PM to 8AM (for lighting condition and distribution of monkeys, see **Supplementary Table S2**). The intensity of illumination was measured by a Benetech GM1020 digital lux meter (Jumaoyuan Inc., Shenzhen, China). Background noise was not intentionally restricted. The room temperature was maintained between 18-30 degrees Celsius.

### 2.4 Food, fruit, and cookie

A standard food pellet was provided twice daily at 9AM and 3PM, each meal lasting for two hours. The amount each monkey ingested was not calculated. Fruits, including apples, bananas, sliced sweet potatoes, cucumbers, carrots, tomatoes, pitaya, and peaches (one kind each time), were provided every other day at 12PM with unlimited access for one hour. Cookies, peanuts, and popcorn were provided irregularly. On days of test and blood sampling (fasting 13-17h) the meal at 9AM would be avoided, but the monkeys had unrestricted access to drinking water, fruit, or their next meal once they woke up from anesthesia.

### 2.5 FBG, HbA1c, insulin, and C-peptide concentration testing

Between 9AM-12PM on each scheduled sampling day, monkeys were anesthetized by receiving an intravenous injection of 1% sodium pentobarbital (50mg/kg) to minimize fluctuations in blood glucose levels, and blood samples were drawn 30 minutes later. The FBG was measured on-site using the Ascensia Breeze Blood Glucose Monitoring System (Newbury, Berks, UK). The acceptable range for FBG levels was set at 1.1-33.3mmol/L. Any concentration found to be below 1.1 was discarded, while any concentration exceeding 33.3 was recorded as 33.3 for statistical analysis.

The plasma concentrations of HbA1c, insulin, and C-peptide were separately evaluated using enzyme-linked immunosorbent assay (ELISA) kits (R&D System, Beijing, China) and analyzed by Huanya Biomedicine Technology Co. LTD (Beijing, China). The results were read using a microplate reader (Multiskan MK3, Thermo Scientific, Beijing, China) at a wavelength of 450nm. Any uncertain HbA1c results, whether they are too high or too low, such as those that cannot be determined due to hemolysis, and results below 2.6%, will be discarded.

### 2.6 Retrospective grouping of monkeys according to glucose metabolic status

For the grouping of monkeys enrolled, we utilized the clinical diagnostic criteria for diabetes that are currently employed in humans (2). Additionally, we took into account the concentration of HbA1c and weight loss as diagnostic criterion, but the latter was evaluated in conjunction with FBG. Based on the data, the monkeys in this study were retrospectively categorized into four groups: i) SDM (Spontaneous Diabetes Mellitus, consisting of monkeys diagnosed with diabetes at the onset of the formal experimental session), ii) LID (Light-Induced Diabetes, consisting of monkeys that developed diabetes during the experimental session), iii) IFG (impaired fasting glucose tolerance, consisting of monkeys with elevated FBG and/or HbA1c level but below diabetes diagnosis standard), and iv) NGT (monkeys with normal glucose tolerance, all the remaining monkeys). The diagnosis of each monkey that died during the experimental session was based on the data already collected by the death. The diagnostic rates were comprehensively listed in **Table 1**.

**Table 1:** The diagnostic criteria and number count of monkeys in this study.

Group	Diagnostic criteria	N*	Rates
SDM	1) FBG≥11.1mmol/L at baseline 2) FBG≥7.0mmol/L and/or HbA1c≥6.5% for consecutive two times starting from baseline	4 7	5.58 (11/197)
LID	1) FBG≥11.1mmol/L once after baseline 2) FBG≥7.0mmol/L non-consecutive twice 3) HbA1c≥6.5% once after baseline 4) 11.1>FBG ≥7.0mmol/L measured once after baseline, with a consistent weight loss for over six months, resulting in a total loss of 10% or more	10 48 16 9	44.62 (83/186)
IFG	7.0mmol/L>FBG≥6.1mmol/L, and/or 6.5>HbA1c≥6.1%, twice or more	36	19.36 (36/186)
NGT	The remaining besides SDM, LID, and IFG	67	36.02 (67/186)

Listed the diagnostic criteria of spontaneous diabetes mellitus, light induced diabetes, and impaired fasting glucose, as well as the final number of monkeys in each group. FBG, fasting blood glucose; HbA1c, glycosylated hemoglobin; IFG, impaired fasting glucose tolerance; LID, light induced diabetes; NGT, normal glucose tolerance; SDM, spontaneous diabetes mellitus. *based on the first reached criteria.

### 2.7 Verify of the diabetes condition

After an intravenous injection of glucose solution, the glucose metabolism condition was tested in 12 randomly selected monkeys from the LID group using the 2hGTT (5g/Kg weight). This test was adapted from the IGT test in humans, which measures blood glucose levels after consuming a standardized amount (75g) of glucose. All 12 monkeys verified positive for diabetes.

### 2.8 Statistical analysis

Raw data were collected for age, weight, gender, concentrations of FBG, HbA1c, insulin, and C-Peptide. All statistical tests in this report were two-tailed. To produce statistical results, Microsoft Excel, SigmaPlot 11.0 and GraphPad InStat 3.10 for Windows were utilized.

By running SigmaPlot one way repeated measures Analysis of Variance (ANOVA) raw data across the entire batch or within a subgroup of monkeys were compared. This was done to determine whether there were any significant differences among various testing time points. Subsequently, the Bonferroni t-test was applied to compare data from each time point against the baseline of the same group. To determine if there were any differences between two subgroups at each time point, raw data were compared using GraphPad InStat 3.10 ordinary One-way ANOVA, followed by Tukey-Kramer Multiple Comparison Test to compare data from one group with other group at the same point, thereby identifying the source of the difference and the significance of its magnitude. The data were presented as mean ± standard division (s.d.). Differences were found to be statistically significant at α=0.05 level. The P-value, F-value (or H-value in case of Kruskal-Wallis One Way ANOVA on Ranks), animal numbers (N), and degrees of freedom (DF) for the ANOVA results were listed in **Supplementary Tables 3**-**6**. The sources of the difference and the significance of the magnitude were displayed in corresponding figures.

The correlation between the brightness at night and concentrations of FBG, HbA1c, insulin, and C-Peptide in whole batch or subgroup of monkeys were calculated in SigmaPlot by running the linear regression, using the median of night brightness of 13, 35 and 75Lux as independent, while concentrations of FBG, HbA1c, insulin, or C-Peptide of each monkey lived in the environments or in LID, IFG, and NGT subgroups were used as dependents. The time course of correlation coefficient between predicted concentrations of FBG and light, age, weight, light plus age, light plus weight, or light plus age plus weight was calculated by running linear or multiple linear regression in SigmaPlot. Median brightness at night was used as the dependent variable, while baseline age and the averaged weight of each monkey during the experiment session were used as independent variables.

## 3 Results

### 3.1 Exposure to light at night deteriorated glucose metabolism in SDM monkeys

**Figure 1:**
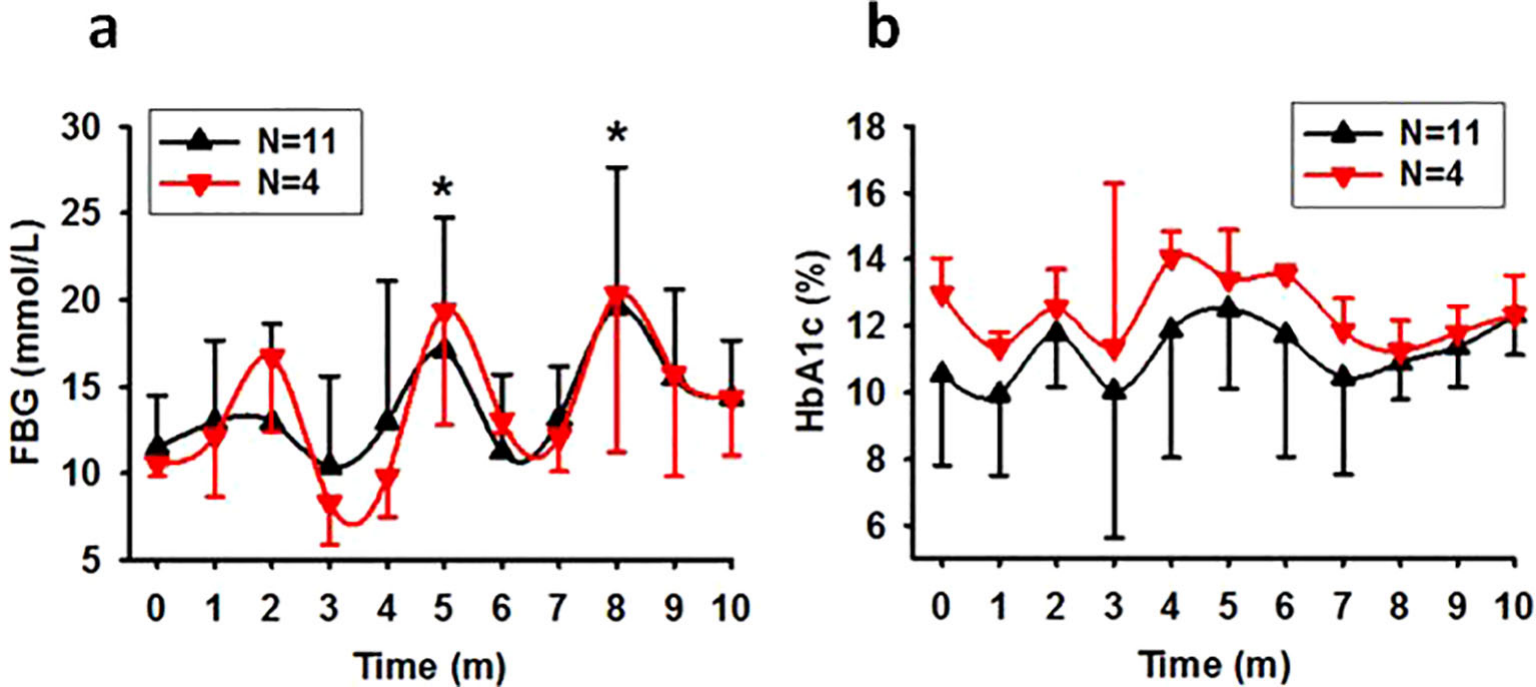
The time course of FBG and HbA1c levels in spontaneous diabetic monkeys. Showing that exposure to nocturnal light exacerbates glucose metabolism in previously diabetic monkeys. **(A)**, Trend of FBG concentration in SDM monkeys (*P<0.05 vs 0 with N=11). **(B)**, Trend of HbA1c concentration in SDM monkeys (P=0.269 with N=11). Time, experimental time in month (m). Error bar, standard division.

The SDM rate was 5.58% (11/197). Exposure to light during night time led to a decline in glucose metabolism ability in this group, evidenced by elevated average FBG concentrations at later time points (**Figure 1A**, N=11, P=0.003; N=4, P=0.009 excluding data from the dead) and high death rate. During the session, 7 of the 11 monkeys died prematurely (63.64%), including 6 from the 75Lux room (1 at month 3, 1 at month 4, 3 at month 6, and 1 at month 9), and 1 from the 13Lux flash neon room at month 6. All the survived monkeys in SDM group (4/11) were from the 13Lux room. By “prematurely” we mean that the animal died before the end of the designed experimental session and that light in the sleeping environment caused the monkeys died earlier than expected in environments without that light.

Despite the high starting HbA1c concentrations observed in most of the monkeys in SDM group, there was slight increase in HbA1c concentrations at one of the later time points for every monkey. However, one-way ANOVA did not reveal statistically significant differences between different time points (**Figure 1B**).

### 3.2 Routine and long-term exposure to bright night intensity-dependently elevated FBG levels in previously healthy monkeys

**Figure 2:**
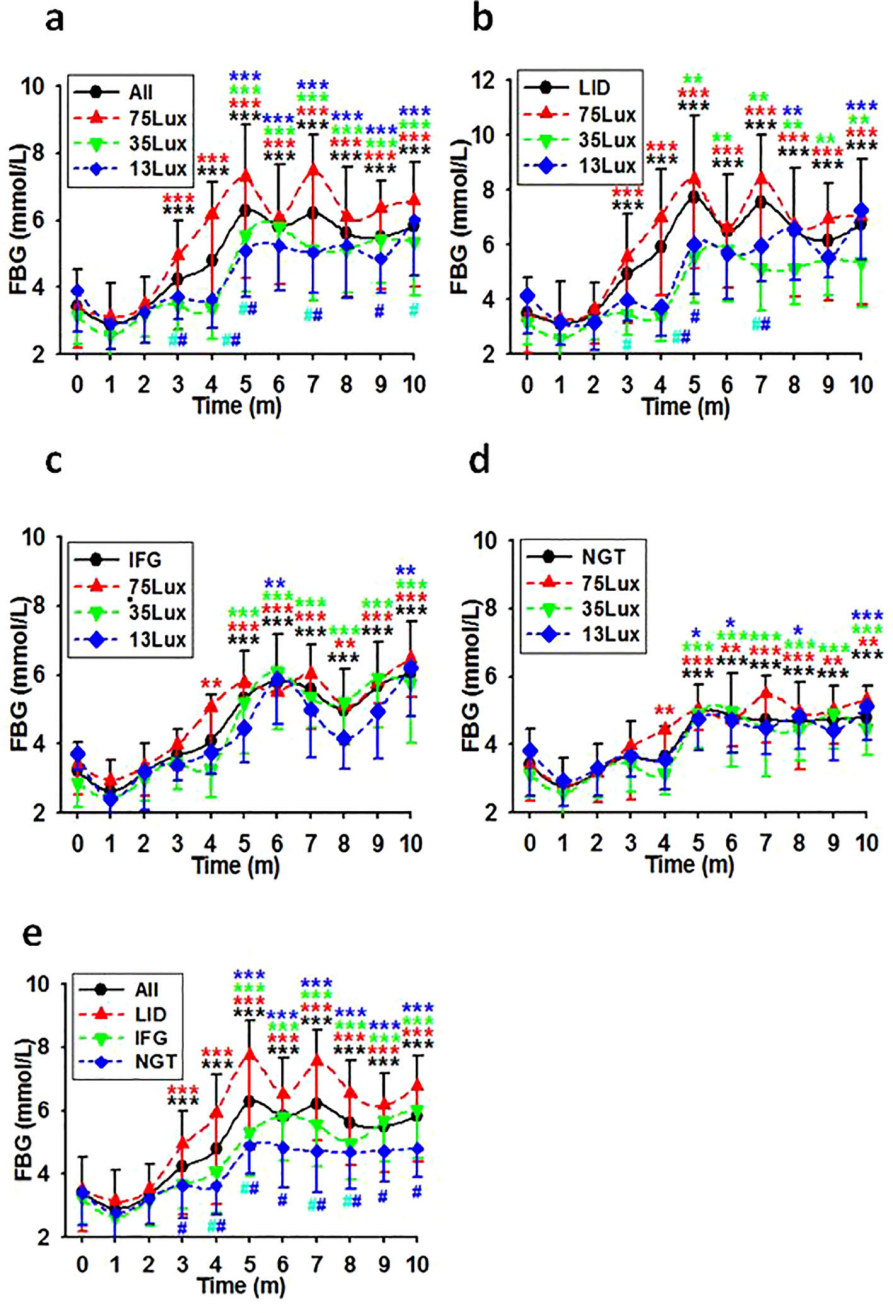
FBG level courses in previously healthy monkeys exposed to light at night. Light exposure intensity and time dependently led to a consistent increase in FBG levels for the initial 5 months, with these levels persisting at elevated states thereafter in overall **(A)**, LID **(B)**, IFG **(C)**, & NGT **(D)** groups. Time, experimental time in months (m); 75, 35, and 13Lux, median light intensities of 75, 35 and 13Lux respectively; *,**,***, P<0.05, P<0.01, P<0.001 vs 0 of the same group, ^#^P<0.05 vs 75Lux or LID group **(E)** at the same time point, respectively and distinguished by different colors. Error bar, standard division.

According to the diagnostic criteria in **Table 1** all monkeys with normal glucose metabolism status upon entering the experiment session (N=186) were counted into LID, IFG, and NGT groups (N=83, 36, and 67 respectively). As detected monthly, the average FBG concentrations in these groups increased gradually during the first five months and remained high in the following months (**Figures 2A-D**). As analyzed for the whole batch as a group (All), exposure to bright night elevated the FBG concentration significantly by the third experimental month. However, if separated, significant differences appeared by the third month in the LID group and by the fifth month in both IFG and NGT groups. Furthermore, as compared with the concentration of FBG in monkeys exposed to 35 and 13Lux light at night, the FBG of monkeys exposed to the 75Lux was significantly higher. However, the FBG concentrations in the IFG and NGT groups showed no significant difference at any time point under any light intensities. Nevertheless, a similar upward trend for FBG was observed in both groups, similar to that seen in overall and LID groups (**Figures 2A-E**).

Linear regression analysis using median light intensities as independent variables and the morbidities of LID, IFG, and LID+IFG in monkeys exposed to corresponding bright night as dependent revealed a significant positive correlation between the light intensities and the morbidities (**Table 2**).

**Table 2:** The correlation between the rate of glucose metabolic disorder and light intensity at night.

	75 Lux	35 Lux	13 Lux	Correlation			
				Formula	R	F	P-value
**LID**	58.7% (54/92)	29.82% (17/57)	32.43% (12/37)	Y=21.831+0.443L	0.904	4.457	0.282
**IFG**	16.3% (15/92)	26.32% (15/57)	16.22% (6/37)	Y=20.814-0.0293L	0.159	0.0258	0.899
**LID+IFG**	75% (69/92)	56.14% (32/57)	48.65% (18/37)	Y=42.273 +0.431L	0.997	163.354	0.05
**NGT**	25% (23/92)	43.86% (25/57)	51.35% (19/37)	Y=57.727 -0.431L	0.997	163.354	0.05

IFG, impaired fasting glucose tolerance; LID, light induced diabetes; NGT, normal glucose tolerance. F, F value; L, light intensity; R, the coefficient of correlation; Y, the predicted glucose metabolic morbidity in response to L.

Further linear regression analysis, which utilized median light intensities as independent variables and the averaged after-baseline FBG concentration of each monkey living in the corresponding environment as the dependent, demonstrated that FBG concentrations were dependent on light intensities in both the LID and IFG groups (**Table 3**). Using factors such as median light intensities, age, body weight, or any combination of them as independent variables and the averaged after-baseline FBG concentration as the dependent, a collection of month-by-month linear regression analysis results were listed in **Table 4** and **Supplementary Table S7**.

**Table 3:** The correlation between FBG and light at night in previously healthy monkeys.

Group (N)	Formula	R	F	P-value
**All (186)**	G=3.973+0.0165L	0.35	25.763	<0.001
**LID (83)**	G=4.372+0.0247L	0.409	16.253	<0.001
**IFG (36)**	G=4.262+0.0078L	0.528	13.122	<0.001
**NGT (67)**	G=4.19+0.0039L	0.142	1.33	0.253

All, whole batch of monkeys; IFG, impaired fasting glucose tolerance; LID, light induced diabetes; NGT, normal glucose tolerance. F, F value; G, the predicted FBG concentration in response to light intensities; L, light intensity; N, animal number; R, the coefficient of correlation.

**Table 4:** The month-by-month correlation between FBG to light, age, weight, or their combination.

Month (N)	FBG-light, Formula (G =)	R	F	P-value
3 (180)	2.928+0.026L	0.369	28	<0.001
4 (177)	2.227+0.0508L	0.538	71.394	<0.001
5 (176)	4.354+0.0385L	0.375	28.515	<0.001
6 (172)	5.243+0.0115L	0.154	4.096	0.045
7 (172)	3.921+0.0461L	0.494	54.488	<0.001
8 (172)	4.76+0.0172L	0.218	8.471	0.004
9 (171)	3.94+0.0479L	0.517	60.051	<0.001
10 (171)	5.336+0.0131L	0.152	3.993	0.047
Month	FBG-age, Formula	R	F	P-value
0 (186)	2.597+0.0633A	0.273	14.866	<0.001
1 (186)	1.854+0.0795A	0.319	20.787	<0.001
2 (180)	2.473+0.0667A	0.331	21.845	<0.001
3 (180)	2.858 +0.105A	0.287	15.959	<0.001
Month	FBG-age-light, Formula	R	F	P-value
0 (186)	2.83+0.0657A-0.0056L	0.3	9.036	<0.001
1 (186)	1.622+0.0772A+0.0051L	0.335	11.602	<0.001
2 (180)	2.271+0.0658A+0.003L	0.348	12.22	<0.001
3 (180)	1.665+0.0994A+0.0251L	0.458	23.457	<0.001
4 (177)	1.601+0.049A+0.0505L	0.548	37.261	<0.001
5 (176)	3.605+0.0584A+0.0382L	0.391	15.607	<0.001
7 (172)	4.789-0.0679A+0.0464L	0.514	30.098	<0.001
8 (172)	4.496+0.0206A+0.0171L	0.224	4.451	0.013
9 (171)	4.682-0.0568A+0.0476L	0.529	32.417	<0.001
Month	FBG-weight, Formula	R	F	P-value
0 (186)	2.667+0.103B	0.201	7.748	0.006
3 (180)	5.339-0.15B	0.186	6.385	0.012
4 (177)	8.08-0.446B	0.417	36.806	<0.001
7 (172)	7.962-0.237B	0.226	9.077	0.003
9 (171)	8.935-0.356B	0.343	22.34	<0.001
Month	FBG-weight-light, Formula	R	F	P-value
0 (186)	2.681+0.102B-0.0002L	0.201	3.854	0.023
1 (186)	1.659+0.0978B+0.0106L	0.203	3.952	0.021
3 (180)	3.086-0.0168B+0.0253L	0.369	13.962	<0.001
4 (177)	4.4-0.231B+0.0415L	0.571	42.16	<0.001
5 (176)	3.505+0.0901B+0.0422L	0.382	14.737	<0.001
6 (172)	3.852+0.147B+0.0175L	0.219	4.23	0.016
7 (172)	3.844+0.0082B+0.0465L	0.494	27.09	<0.001
8 (172)	4.09+0.0707B+0.0201L	0.229	4.675	0.011
9 (171)	5.247-0.137B+0.0417L	0.528	32.279	<0.001
Month	FBG-age-weight-light, Formula	R	F	P-value
0 (186)	1.91+0.0648A+0.0985B-0.0014L	0.344	8.128	<0.001
1 (186)	0.751+0.0763A+0.0932B+0.0091L	0.367	9.418	<0.001
2 (180)	2.101+0.0653A+0.0187B+0.005L	0.35	8.207	<0.001
3 (180)	1.964+0.1A-0.0329B+0.0238L	0.459	15.647	<0.001
4 (177)	3.778+0.0555A-0.24B+0.0408L	0.583	29.62	<0.001
5 (176)	2.871+0.0562A+0.0809B+0.0415L	0.396	10.652	<0.001
6 (172)	3.727+0.0115A+0.145B+0.0147L	0.221	2.858	0.039
7 (172)	4.598-0.0686A+0.0211B+0.0473L	0.514	19.978	<0.001
8 (172)	3.889+0.0185A+0.0671B+0.0199L	0.233	3.227	0.024
9 (171)	5.818-0.0526A-0.126B+0.0424L	0.54	22.722	<0.001

Linear regression analysis results showing that the FBG concentrations could be affected by age, weight, light, and their combination. A, age; B, weight; F, F value; FBG, concentration in response to light intensity; G, the predicted FBG concentration; L, light; R, the coefficient of correlation. Lines with P>0.05 have been removed; refer to **Supplementary Table S7** for the complete table.

### 3.3 Differential change patterns of HbA1c with FBG upon exposure to light at night in previously healthy monkeys

**Figure 3:**
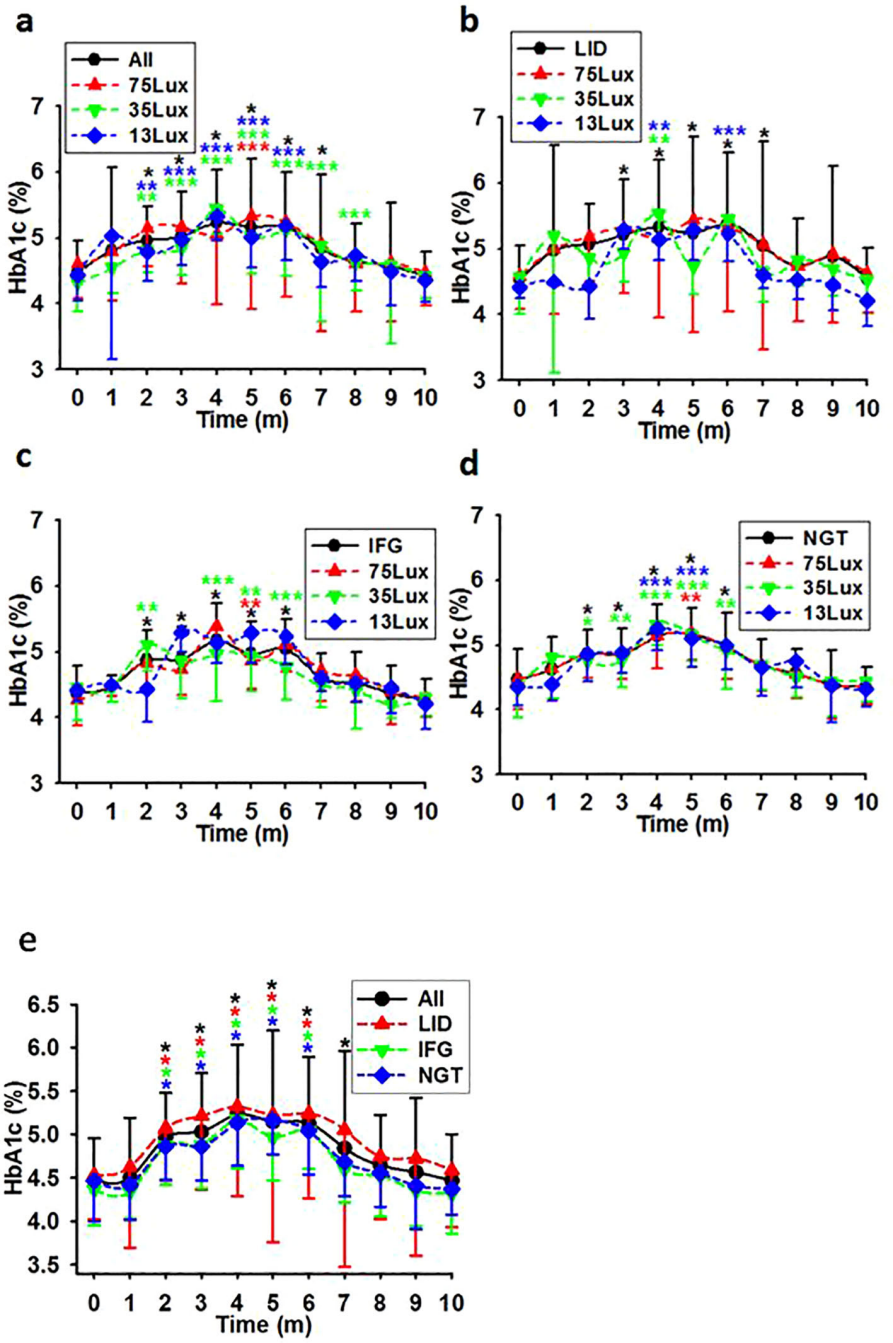
HbA1c level courses in previously healthy monkeys exposed to light at night. Light exposure led to a comparable elevation in HbA1c levels for the first 4 months, followed by a steady decline until ultimately approaching normal levels in overall **(A, E)**, LID **(B, E)**, IFG **(C, E)**, & NGT **(D, E)** groups. Time, experimental time in months (m); 75, 35, and 13Lux, median light intensities of 75, 35 and 13Lux respectively; *,**,***, P<0.05, P<0.01, P<0.001 vs 0 of the same group respectively and distinguished by different colors. Error bar, standard division.

In contrast to FBG, using median light intensities as independent variables and the averaged after-baseline HbA1c concentration of each monkey living in the corresponding environment as the dependent, linear regression analysis did not reveal any notable disparities in the average HbA1c concentrations among the monkey groups. Further calculation revealed a moderate correlation between light intensity and the HbA1c concentrations at experimental months 3,4, and 5. The correlation coefficients were R=0.321 (P<0.001, N=179), 0.171 (P=0.022, N=179), and 0.195 (P=0.009, N=177) for the whole batch. These coefficients suggest a correlation between HbA1c and light at night during months 3-5 (**Figures 3A-D**). However, when plotted into time course, there was no significant difference between any two lines representing HbA1c levels at any time point. (**Figures 3A-E**).

### 3.4 Light induced diabetes as reflected by insulin and C-peptide production

**Figure 4:**
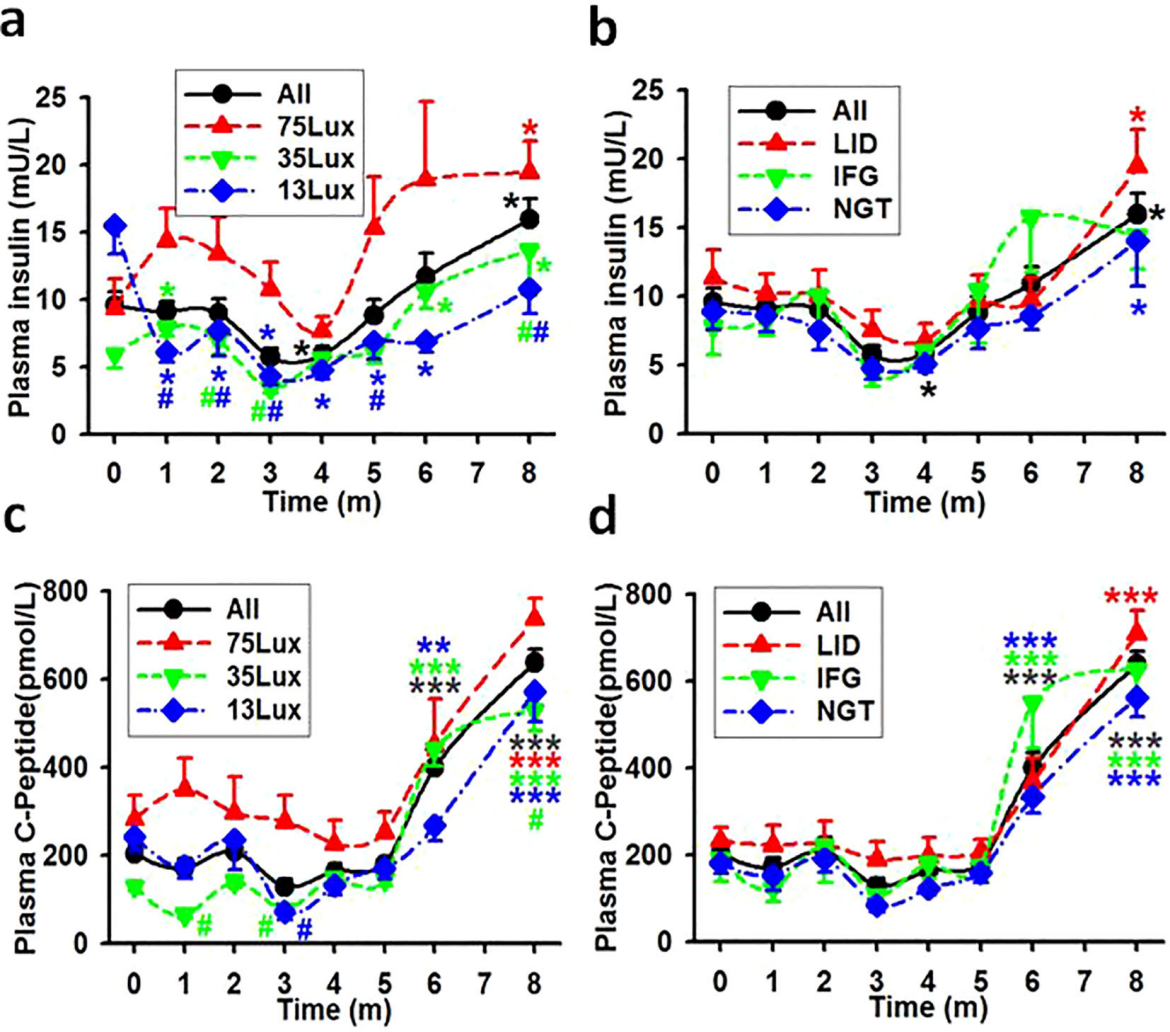
The average concentrations of fasting insulin and C-peptide in monkeys. Showing the average concentrations of fasting insulin **(A, B)** and C-peptide **(C, D)** in plasma during experimental month 0-8. *, **, ***, P<0.05, P<0.01, P<0.001 vs baseline (0) of the same group, ^#^P<0.05 vs 75Lux at the same time point, respectively and distinguished by different colors. Error bar, standard division.

The one-way ANOVA and the time course of fasting insulin concentrations revealed that: 1) Stronger night brightness had stronger impact on the production of insulin during the experimental session (**Figure 4A**). There were statistical differences between the concentrations of insulin in monkeys exposed to 75Lux and 35 or 13Lux at multiple points. However, no statistical differences found among LID, IFG, and NGT monkey groups (**Figure 4B**). 2) The insulin concentration would decline slowly for the first three months and then elevated to a statistical higher level (**Figures 4A, B**). 3) The line depicting the average insulin levels for LID monkeys was higher than that of the IFG and NGT groups at most of the points (**Figure 4B**).

The estimation of insulin production can be more precisely determined by measuring plasma C-peptide levels (18). The plasma C-peptide levels in monkey groups of the entire batch (All), 75Lux, 35Lux, and 13Lux, as well as in LID, IFG, and NGT were all statistically higher by month 6-8 than before the start of the experiment (**Figures 4C, D**). When considering light intensity as a standalone factor, positive correlation coefficients were resulted between the concentrations of insulin/C-Peptide and the light intensities in all monkey groups (**Table 5**).

**Table 5:** The correlation between the average fasting insulin/C-peptide concentrations in plasma and brightness at night.

Group	Formula	R	F	P-value	DF
All (186)	I=2.585+0.212L	0.309	19.272	<0.001	185
LID (83)	I=3.954+0.221L	0.23	4.504	0.037	82
IFG (36)	I=3.671+0.118L	0.54	13.998	<0.001	35
NGT (67)	I=4.271+0.112L	0.356	9.425	0.003	66
All (186)	C=50.23 +7.118L	0.486	56.751	<0.001	185
LID (83)	C=38.284+8.119L	0.438	19.202	<0.001	82
IFG (36)	C= 114.374+5.639L	0.425	7.489	0.01	35
NGT (67)	C=76.126+5.279L	0.557	29.164	<0.001	67

Linear regression analysis results showing the predicted fasting insulin/C-Peptide concentrations in response to light. All, whole batch of monkeys; C, C-peptide; DF, degree of freedom; F, F value; I, insulin; IFG, impaired fasting glucose tolerance; L, light intensity; LID, light induced diabetes; NGT, normal glucose tolerance; R, the coefficient of correlation.

### 3.4 The involvement of age, weight, and sex in the development of glucose metabolic disorders

#### 3.4.1 Weight

**Figure 5:**
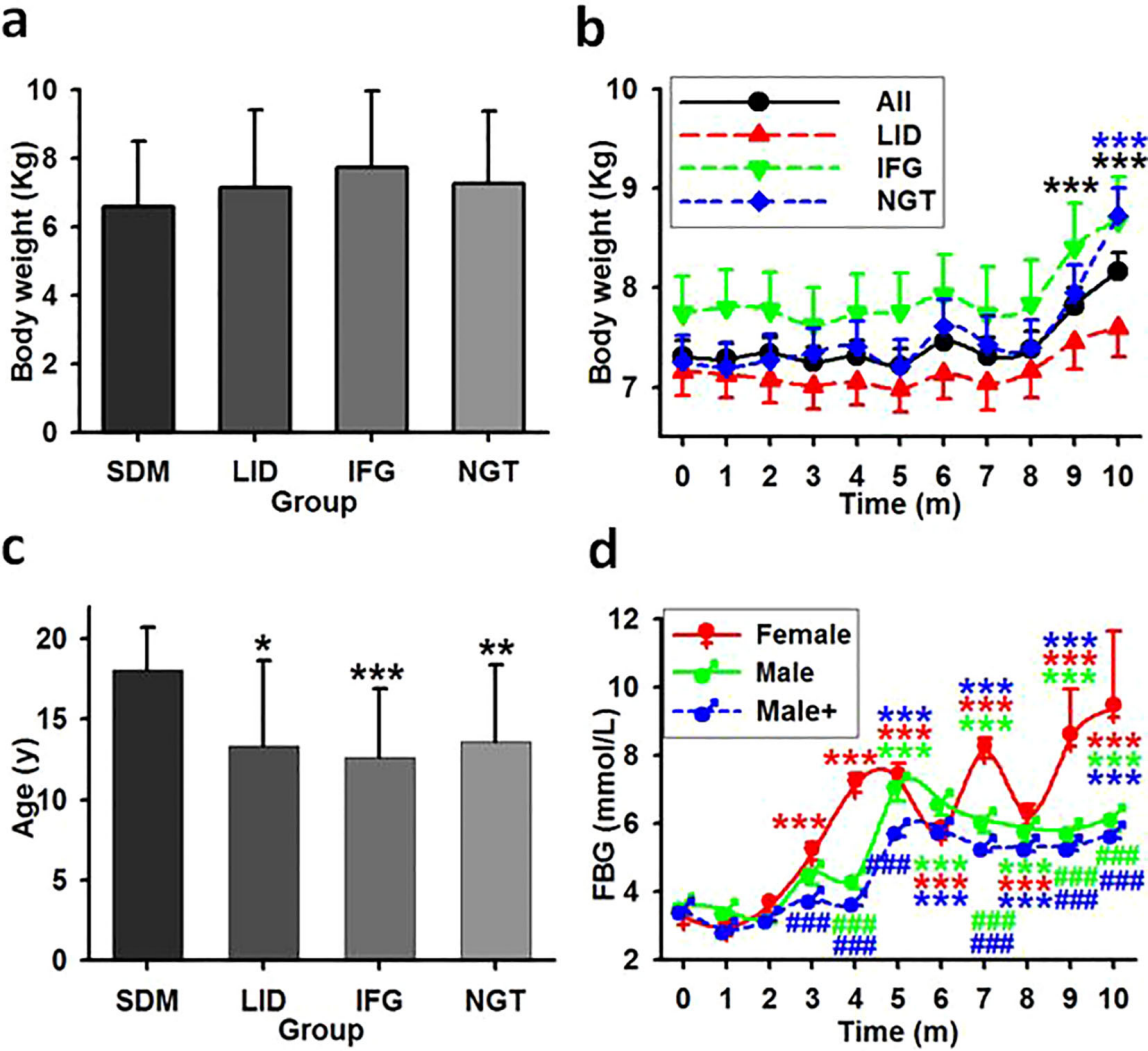
The impact effect of age, weight, and gender in glucose metabolism disorder. **(A)** Averaged weight in monkey groups. Showing that no significant statistical differences exist among the groups. **(B)** The time course of the average weight in previously healthy monkeys. Showing that weight statistically increased for overall and NGT group but not LID and IFG group. ***, P<0.001 vs time point 0. **(C)** Averaged age in monkey groups. Showing that age is associated with the occurrence of spontaneous diabetes cases but not with new cases. *,**,***, P<0.05, P<0.01, P<0.001 vs SDM group. **(D)** FBG concentrations in female and male monkeys. Male, monkeys living in the same environment (75Lux) with female; Male+, all males in LID, IFG and NGT groups. ***P<0.001 vs time point 0 of the same group, ^###^P<0.001 vs female at the same time point, respectively and distinguished by different colors.

**Figure 6:**
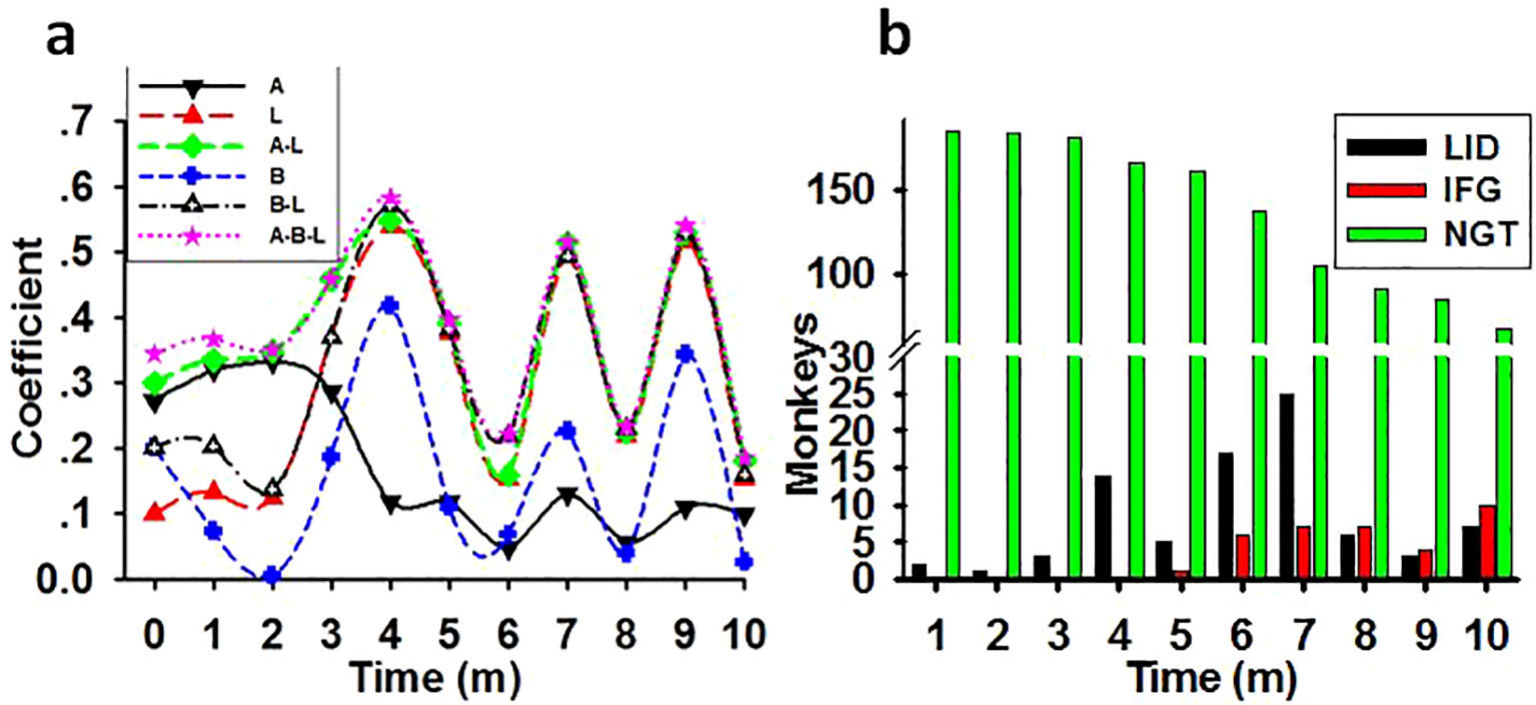
The synergistic impairing effect of light with age and weight on glucose metabolism. **(A)** The time course of the correlation coefficient between the impairing factors and the monthly tested FBG concentrations in previously healthy monkeys (N=186). Showing the possible impairing ability of light at night (L), age **(A)**, weight **(B)**, or combination of age and light (A-L), weight and light (B-L), and age plus weight plus light (A-B-L) on glucose metabolism in monkeys. **(B)** Increasing monkey number of LID and IFG cases and decreasing of NGT number during the experimental session. Coefficient, correlation coefficient between impact factors and FBG concentrations; Time, experimental time in months (m).

One way repeated ANOVA revealed a slight disparity and the starting weights seemed lower for the SDM group (**Figure 5A**, F=3.094, P=0.03, N=197). No significant difference were found between the average weights of SDM and any one of the previously healthy monkey subgroups LID, IFG, and NGT (**Figure 5B**, ANOVA, F=1.362, P=0.096, N=186). Every group had weight gain trends with statistical differences emerged only in NGT group compared between the initial average weight and weight at month 10 (**Figure 5B**, ANOVA, F=2.643, P=0.0049, N=186). Eight monkeys in the LID group lost weight for over 6 consecutive months and lost 10% or more in total. Regression analysis revealed fluctuating correlation between the starting weight of each monkey and the monthly tested FBG (**Table 4**, **Figure 6A**).

#### 3.4.2 Age

Averagely the SDM group had a statistically older age (mean 18.09, s.d. ± 2.63) than LID and IFG groups (ANOVA on Ranks, H=9.686, P=0.021). However, there was no significant differences among the LID, IFG, and NGT groups (**Figure 5C**, repeated one-way ANOVA, F=0.079, P=0.924, N=186). Regression analysis revealed a moderate correlation between the age of each monkey and its monthly tested FBG levels for the first three experimental months (**Table 4**, **Figure 6A**).

#### 3.4.3 Gender

Female monkeys excluding that from SDM group were housed in rooms with a median night brightness of 75Lux (N=62). The morbidities of LID and IFG of these animals were 58.06% and 14.52%, which were no difference as compared to that of the males under this brightness (N=30) (t=0, P=1, **Table 6**). One-way ANOVA revealed statistical difference (P=0.031) between the average after-baseline FBG concentrations of females (mean 5.734, s.d. ± 1.8, N=62) and male living exclusively in this environment (5.33 ± 1.31, N=30). The differences appeared at month 4, 6, 7, 9 and 10. Significantly higher FBG level in females appeared from experimental month 3 while from month 5 in males. With brightness at night as independent, one-way ANOVA revealed significant difference between the averaged FBG concentrations of female (mean 5.734, s.d. ± 1.8, N=62) and male (Male+, mean 4.67, s.d. ± 0.93, N=124) and significant differences between genders appeared earlier from month 3 (F=67.166, P<0.001, N=186, **Figure 5D**).

**Table 6:** Differential morbidity between genders in previously healthy monkeys.

Gender (N)	Light intensity (N)	LID (%)	IFG (%)	NGT (%)
Male (124)	75 Lux (30)	60 (18/30)	20 (6/30)	30 (6/30)
	35 Lux (57)	29.82 (17/57)	26.32 (15/57)	43.86 (25/57)
	13 Lux (37)	32.43 (12/37)	16.22 (6/37)	51.35 (19/37)
Female (62)	75 (62)	58.06 (36/62)	14.52 (9/62)	27.42 (17/62)

Showing the differential morbidities of LID and IFG between male and female. IFG, impaired fasting glucose tolerance; LID, light induced diabetes; NGT, normal glucose tolerance; R, the coefficient of correlation.

### 3.5 The synergistic effect of light at night, age, and weight in the development of glucose metabolic disorders

As shown in **Table 4**, there was weak correlation between weight or age and the FBG level before the start of the experiment. Nevertheless, multiple linear regressions revealed a synergistic effect of light with both age and weight in impairing glucose metabolism in previously healthy monkeys. Time course lines representing the correlation coefficient between the monthly tested FBG concentrations and factors such as light, age, weight, or any combination of these factors were displayed in **Figure 6A**. In parallelism with **Table 4**, the coefficients in **Figure 6A** showed that, before the start of the experiment light was the lowest risk as a single impacting factor on glucose metabolism, with weight in the middle and age had more powerful impact. Starting from experimental month 3 the impairing effect of light at night climbed to a high level and acted as an aggravator to both age and weight on glucose metabolism disorders.

A monthly counting of LID, IFG, and NGT monkeys was shown in **Figure 6B**. The bars demonstrated that, as regular exposure to the experimental environment prolonged, the incidence of diabetes and IFG cases increased while number in NGT group shrunk.

## 4 Discussion

In this study, we manipulated the lighting conditions in sleeping quarters for monkeys over a period of 10 months. The daytime brightness was designed to replicate that experienced by urban populations at the Tropic of Cancer, while the dark hours were illuminated using three different intensities of light. The results revealed a significantly high mortality rate among monkeys that entered the experimental phase with diabetes, as well as a notable increase in the incidence of diabetes and IFG in previously healthy individuals. Given that only nighttime brightness was altered from typical circadian rhythms and that consistent all-day bright living conditions were maintained throughout the study duration, we propose that exposure to bright light at night, rather than sleep duration or circadian rhythm per se, was a critical factor contributing to disturbances in glucose metabolism. This effect appeared to be dependent on light intensity, as evidenced by an elevated death rate among diabetic monkeys and an accelerated rise and higher levels of FBG observed in previously healthy monkeys housed under the most intense nighttime lighting conditions.

Both overweight and age are well-established risks of T2D (19, 20). However, in the current study, there were no statistically significant differences in the average starting weight among the SDM, LID, IFG, and NGT monkey groups. Although the time course representing the monthly averaged weight of the LID group consistently remained below that of both the IFG and NGT groups, no significant differences were observed at any time point. Notably, when compared to their initial average weights, only the NGT group exhibited a statistically significant increase in weight at month 10. Conversely, it was found that the average age of the SDM group was greater than that of previously healthy monkey groups. When excluding the SDM group from analysis, a month-by-month evaluation revealed that weight acted as an inconstant impairing factor on FBG levels. While age served as a moderate impairing factor for FBG during the first three months, its impact dramatically diminished after month four and remained low thereafter. Nevertheless, correlations between FBG and combinations of weight and/or age increased to moderate levels at months four, seven, and nine. These findings indicate that exposure to bright light at night adversely affects glucose metabolism not only as an isolated factor but also acts synergistically with age and weight. Additionally, female gender emerged as another variable potentially influenced by nighttime light exposure. Although male and female subjects displayed comparable morbidity rates for LID and IFG under identical bright sleeping conditions, females tended to exhibit higher FBG levels, an effect which manifested two months earlier than in males.

Based on the ELISA results of insulin and C-Peptide concentrations it was obvious that monkeys exposed to 75Lux nocturnal light or those in the LID group exhibited higher insulin production compared to monkeys exposed to 35 and 13Lux, or those in the IFG and NGT groups. Despite this, the FBG levels in monkeys of LID, IFG, and NGT groups increased over the course of the experiment. These findings suggest that, after prolonged exposure to the bright sleeping environment at night, monkeys developed insulin resistance. Eventually, the insulin production was unable to meet the glucose metabolism demands, leading to a disruption in homeostasis and subsequently resulting in IFG and T2D.

It can be seen that nighttime light is an important damaging factor of glucose metabolism. Primates exposed to a bright sleeping environment may experience a reduction in the duration of the nighttime sleep, an increase in the frequency of arousals and awakenings after the onset of sleep, and other disturbances that can usually be avoided during the dark phase of their natural circadian rhythm. These happenings may disrupt the production and secretion of melatonin, a hormone that plays a role in timing circadian rhythms (21), and regulates glucose metabolism (22) through receptor-dependent influences on glucagon and insulin secretion (23, 24). In situations such as misaligned circadian rhythms or reduced sleep duration insulin sensitivity also decreases (25).

Evidences show that both insulin and glucagon secretion are modulated by melatonin but at different times and rates. In pancreatic islets, the melatonin receptor type 1 (MT1) is expressed on α-cells while type 2 (MT2) on β-cells (22, 26). Once secreted, melatonin increases glucagon production from α-cells immediately (27) but inhibits insulin production in β-cells (28) with a functional phase-shift (29). In the case of T2D, insulin secretion may lose a portion of its negative regulatory mechanism, leading to hyperinsulinemia (30). This may be the reason for the concurrent hyperglycemia and hyperinsulinemia in individuals with T2D (31).

HbA1c, glycosylated hemoglobin that serves as an indicator of the average blood glucose level over a period ranging from weeks to months, didn’t show fluctuant trend as the FBG in this study. Instead, the elevation in HbA1c levels reached its peak at month 4 and subsequently returned to a level comparable to the baseline, unlike the consistently increasing concentrations of FBG during the initial five months. Additionally, when compared to the baseline, the concentrations of HbA1c in the overall, IFG, and NGT groups were significantly elevated starting at month 2, which appeared earlier than the significant mark for FBG. Surprisingly, the time course line reflecting the average HbA1c in LID, IFG, and NGT groups in this study were parallel all the way. Considering that HbA1c reflects the average blood glucose levels rather than real-time concentrations, all the above phenomena may indicate that, upon exposure to light at night, the blood glucose concentrations were significantly elevated in the initial months following meals, and that HbA1c may be not a proper indicator of T2D by itself.

Over the past few decades, the world has experienced a significant surge in the number of individuals with diagnosed diabetes and undiagnosed IFG (32). Despite desperate efforts, attempts to curb this rise have proven ineffective (32, 33). The current study demonstrates that the onset of diabetes and IFG in the monkeys is closely related to the experimental light condition, especially the intensity of the brightness at night.

Accumulating references indicate that, the incidence of T2D and IGT in urban areas is higher than in rural areas (4, 5, 32).The experimental illuminations in this study mimicked that in modern cities, such as the bright night and the artificial circadian. The results could potentially provide valuable insight into the escalating global rates of diabetes and IGT, particularly in industrialized regions. To some extent, a comparison between a satellite map of Earth at night from NASA (2008) (34), which represents the brightness of the world at night and possibly also the urbanization degree of an area, and the world diabetes prevalence map (2007) (32), which shows the prevalence of glucose metabolism disorders, reveals a more explainable causal link between the brightness at night and the prevalence of diabetes. This is to say, the brighter an area the higher the diabetes morbidity. Predicted from the monthly increasing number of LID and IFG, the decreasing number of NGT in this study, taking into account the global population size, and the rapid pace of urbanization, it is possible to explain and anticipate the constantly increasing prevalence of diabetes and IGT of recent years.

## 5 Conclusion

This research demonstrates that exposure to light at night is associated with an increased mortality rate among individuals with pre-existing diabetes, and that light exposure leads to a higher incidence of diabetes and IGT in previously healthy primates. Furthermore, nocturnal light exacerbates issues related to glucose metabolism in conjunction with aging and body weight. In summary, nighttime illumination is closely linked to the significant rise in T2D and IGT observed over the past few decades. We also propose that impaired glucose metabolism may be a secondary consequence of disrupted melatonin secretion resulting from light exposure.

## Data Availability

The raw data supporting the conclusions of this article will be made available by the authors, without undue reservation.
