# Supplementary material for: Bright night sleeping environment induces diabetes and impaired glucose tolerance in non-human primates
Source: Front Endocrinol (Lausanne). 2025 Feb 12;16:1454592. doi: 10.3389/fendo.2025.1454592 (PMC11860132; doi:10.3389/fendo.2025.1454592)
Supplement: Supplementary file 1 [file Table1.docx]

**Supplementary Table 1. Monkey groups based on age.**

| **Age** | **N** |
| --- | --- |
| 5 | 1 |
| 6 | 7 |
| 7 | 7 |
| 8 | 12 |
| 9 | 24 |
| 10 | 17 |
| 11 | 31 |
| 12 | 5 |
| 13 | 5 |
| 14 | 3 |
| 15 | 5 |
| 16 | 10 |
| 17 | 14 |
| 18 | 13 |
| 19 | 13 |
| 20 | 10 |
| 21 | 12 |
| 22 | 3 |
| 23 | 3 |
| 24 | 2 |
| **Total** | **197** |
