# Supplementary material for: Bright night sleeping environment induces diabetes and impaired glucose tolerance in non-human primates
Source: Front Endocrinol (Lausanne). 2025 Feb 12;16:1454592. doi: 10.3389/fendo.2025.1454592 (PMC11860132; doi:10.3389/fendo.2025.1454592)
Supplement: Supplementary file 2 [file Table2.docx]

**Supplementary Table 2.** **Lighting condition and distribution of monkeys.**

| **Brightness (Lux) and duration (hour)** | | | | **N/Sex** |
| --- | --- | --- | --- | --- |
| **Daytime** | | **Nocturnal (median)** | |  |
| 100-200 | 9h | 51-100 white (75) | 15h | 67F, 36M |
| 100-200 | 9h | 21-50 light-blue (35) | 15h | 57M |
| 100-200 | 9h | 6-20 flashing neon (13) | 15h | 37M |
| **Total** | | | | **197 (67F, 130M)** |

|  |  |  |  |  |
| --- | --- | --- | --- | --- |
|  |  |  |  |  |
|  |  |  |  |  |
|  |  |  |  |  |
