# Supplementary material for: Bright night sleeping environment induces diabetes and impaired glucose tolerance in non-human primates
Source: Front Endocrinol (Lausanne). 2025 Feb 12;16:1454592. doi: 10.3389/fendo.2025.1454592 (PMC11860132; doi:10.3389/fendo.2025.1454592)
Supplement: Supplementary file 3 [file Table3.docx]

**Supplementary Table 3. One-way ANOVA results of FBG in monkeys.**

|  | | **P-value** | **F** | **DF** | |
| --- | --- | --- | --- | --- | --- |
|  |  |  |  | **Between months** | **Within months** |
| All (186) | | <0.001 | 121.043 | 10 | 1832 |
| 75 Lux (92) | | <0.001 | 65.681 | 10 | 842 |
| 35 Lux (57) | | <0.001 | 61.876 | 10 | 675 |
| 13 Lux (37) | | <0.001 | 29.506 | 10 | 388 |
| LID  (83) | LID | <0.001 | 65.827 | 10 | 821 |
|  | 75 Lux (54) | <0.001 | 47.839 | 10 | 511 |
|  | 35 Lux (17) | <0.001 | 16.322 | 10 | 163 |
|  | 13 Lux (12) | <0.001 | 12.862 | 10 | 122 |
| IFG (36) | IFG | <0.001 | 38.8 | 10 | 376 |
|  | 75 Lux (15) | <0.001 | 14.94 | 10 | 146 |
|  | 35 Lux (15) | <0.001 | 20.998 | 10 | 163 |
|  | 13 Lux (6) | <0.001 | 7.174 | 10 | 65 |
| NGT (67) | NGT | <0.001 | 42.527 | 10 | 633 |
|  | 75 Lux (23) | <0.001 | 11.958 | 10 | 165 |
|  | 35 Lux (25) | <0.001 | 22.626 | 10 | 267 |
|  | 13 Lux (19) | <0.001 | 13.402 | 10 | 199 |
