# Supplementary material for: Bright night sleeping environment induces diabetes and impaired glucose tolerance in non-human primates
Source: Front Endocrinol (Lausanne). 2025 Feb 12;16:1454592. doi: 10.3389/fendo.2025.1454592 (PMC11860132; doi:10.3389/fendo.2025.1454592)
Supplement: Supplementary file 4 [file Table4.docx]

**Supplementary Table 4. One-way ANOVA results of HbA1c in monkeys.**

| **Group (N)** | | **P-value** | **F** | **DF** | |
| --- | --- | --- | --- | --- | --- |
|  |  |  |  | **Between months** | **Within months** |
| All (186) | | <0.001 | 37.017 | 10 | 1782 |
| 75 Lm (92) | | <0.001 | 11.172 | 10 | 704 |
| 35 Lm (57) | | <0.001 | 14.045 | 10 | 571 |
| 13 Lm (37) | | <0.001 | 16.268 | 10 | 366 |
| LID  (83) | LID | <0.001 | 9.535 | 10 | 790 |
|  | 75 Lux (54) | <0.001 | 5.693 | 10 | 442 |
|  | 35 Lux (17) | 0.006 | 2.597 | 10 | 174 |
|  | 13 Lux (12) | <0.001 | 3.633 | 10 | 122 |
| IFG (36) | IFG | <0.001 | 15.645 | 10 | 336 |
|  | 75 Lux (15) | <0.001 | 4.37 | 10 | 124 |
|  | 35 Lux (15) | <0.001 | 11.053 | 10 | 151 |
|  | 13 Lux (6) | <0.001 | 4.51 | 10 | 66 |
| NGT (67) | NGT | <0.001 | 31.79 | 10 | 680 |
|  | 75 Lux (23) | <0.001 | 8.084 | 10 | 213 |
|  | 35 Lux (25) | <0.001 | 17.537 | 10 | 266 |
|  | 13 Lux (19) | <0.001 | 18.379 | 10 | 199 |
