# Supplementary material for: Bright night sleeping environment induces diabetes and impaired glucose tolerance in non-human primates
Source: Front Endocrinol (Lausanne). 2025 Feb 12;16:1454592. doi: 10.3389/fendo.2025.1454592 (PMC11860132; doi:10.3389/fendo.2025.1454592)
Supplement: Supplementary file 5 [file Table5.docx]

**Supplementary Table 5. One-way ANOVA results of insulin in monkeys.**

|  | **P-value** | **H** | **DF** | |
| --- | --- | --- | --- | --- |
|  |  |  | **Between months** | **Within months** |
| All (186) | <0.001 | 93.852 | 7 | 1054 |
| 75 Lm (92) | <0.001 | 26.861 | 7 | 327 |
| 35 Lm (57) | <0.001 | 56.988 | 7 | 428 |
| 13 Lm (36) | <0.001 | 47.17 | 7 | 271 |
| LID (83) | <0.001 | 39.872 | 7 | 413 |
| IFG (36) | <0.001 | 27.714 | 7 | 224 |
| NGT (67) | <0.001 | 32.953 | 7 | 419 |
