# Supplementary material for: Bright night sleeping environment induces diabetes and impaired glucose tolerance in non-human primates
Source: Front Endocrinol (Lausanne). 2025 Feb 12;16:1454592. doi: 10.3389/fendo.2025.1454592 (PMC11860132; doi:10.3389/fendo.2025.1454592)
Supplement: Supplementary file 6 [file Table6.docx]

**Supplementary Table 6. One-way ANOVA results of C-Peptide in monkeys.**

|  | **P-value** | **F** | **DF** | |
| --- | --- | --- | --- | --- |
|  |  |  | **Between groups** | **Within groups** |
| All (186) | <0.001 | 70.365 | 7 | 1072 |
| 75 Lux (92) | <0.001 | 7.499 | 7 | 325 |
| 35 Lux (57) | <0.0001 | 57.898 | 7 | 452 |
| 13 Lux (36) | 0.003 | 18.994 | 7 | 293 |
| LID (83) | <0.001 | 22.887 | 7 | 413 |
| IFG (36) | <0.001 | 14.575 | 7 | 229 |
| NGT (67) | <0.001 | 34.263 | 7 | 422 |
