# Supplementary material for: Bright night sleeping environment induces diabetes and impaired glucose tolerance in non-human primates
Source: Front Endocrinol (Lausanne). 2025 Feb 12;16:1454592. doi: 10.3389/fendo.2025.1454592 (PMC11860132; doi:10.3389/fendo.2025.1454592)
Supplement: Supplementary file 7 [file Table7.docx]

**Supplementary Table 7. The month-by-month correlation between FBG to light, age, weight, or their combination.**

| **Month (N)** | **FBG-light, Formula (G =)** | **R** | **F** | **P-value** |
| --- | --- | --- | --- | --- |
| 0 (186) | 3.649-0.0045L | 0.0993 | 1.832 | 0.178 |
| 1 (186) | 2.585+0.0064L | 0.132 | 3.249 | 0.073 |
| 2 (180) | 3.106+0.0048L | 0.124 | 2.766 | 0.098 |
| 3 (180) | 2.928+0.026L | 0.369 | 28 | <0.001 |
| 4 (177) | 2.227+0.0508L | 0.538 | 71.394 | <0.001 |
| 5 (176) | 4.354+0.0385L | 0.375 | 28.515 | <0.001 |
| 6 (172) | 5.243+0.0115L | 0.154 | 4.096 | 0.045 |
| 7 (172) | 3.921+0.0461L | 0.494 | 54.488 | <0.001 |
| 8 (172) | 4.76+0.0172L | 0.218 | 8.471 | 0.004 |
| 9 (171) | 3.94+0.0479L | 0.517 | 60.051 | <0.001 |
| 10 (171) | 5.336+0.0131L | 0.152 | 3.993 | 0.047 |
| **Month** | **FBG-age, Formula** | **R** | **F** | **P-value** |
| 0 (186) | 2.597+0.0633A | 0.273 | 14.866 | <0.001 |
| 1 (186) | 1.854+0.0795A | 0.319 | 20.787 | <0.001 |
| 2 (180) | 2.473+0.0667A | 0.331 | 21.845 | <0.001 |
| 3 (180) | 2.858 +0.105A | 0.287 | 15.959 | <0.001 |
| 4 (177) | 4.03+0.0573A | 0.118 | 2.459 | 0.119 |
| 5 (176) | 5.463+0.063A | 0.118 | 2.471 | 0.118 |
| 6 (172) | 5.583+0.018A | 0.0469 | 0.373 | 0.542 |
| 7 (172) | 7.02-0.0622A | 0.13 | 2.883 | 0.091 |
| 8 (172) | 5.315+0.023A | 0.0567 | 0.548 | 0.46 |
| 9 (171) | 6.975+0.0521A | 0.11 | 2.072 | 0.152 |
| 10 (171) | 5.413+0.044A | 0.0992 | 1.679 | 0.197 |
| **Month** | **FBG-age-light, Formula** | **R** | **F** | **P-value** |
| 0 (186) | 2.83+0.0657A-0.0056L | 0.3 | 9.036 | <0.001 |
| 1 (186) | 1.622+0.0772A+0.0051L | 0.335 | 11.602 | <0.001 |
| 2 (180) | 2.271+0.0658A+0.003L | 0.348 | 12.22 | <0.001 |
| 3 (180) | 1.665+0.0994A+0.0251L | 0.458 | 23.457 | <0.001 |
| 4 (177) | 1.601+0.049A+0.0505L | 0.548 | 37.261 | <0.001 |
| 5 (176) | 3.605+0.0584A+0.0382L | 0.391 | 15.607 | <0.001 |
| 6 (172) | 5.04+0.016A+0.0114L | 0.159 | 2.189 | 0.115 |
| 7 (172) | 4.789-0.0679A+0.0464L | 0.514 | 30.098 | <0.001 |
| 8 (172) | 4.496+0.0206A+0.0171L | 0.224 | 4.451 | 0.013 |
| 9 (171) | 4.682-0.0568A+0.0476L | 0.529 | 32.417 | <0.001 |
| 10 (171) | 4.793+0.0425A+0.0129L | 0.18 | 2.799 | 0.064 |
| **Month** | **FBG-weight, Formula** | **R** | **F** | **P-value** |
| 0 (186) | 2.667+0.103B | 0.201 | 7.748 | 0.006 |
| 1 (186) | 2.618+0.402B | 0.0726 | 0.976 | 0.325 |
| 2 (180) | 3.364-0.0022B | 0.0049 | 0.0042 | 0.948 |
| 3 (180) | 5.339-0.15B | 0.186 | 6.385 | 0.012 |
| 4 (177) | 8.08-0.446B | 0.417 | 36.806 | <0.001 |
| 5 (176) | 7.232-0.128B | 0.11 | 2.148 | 0.145 |
| 6 (172) | 5.4+0.0564B | 0.0672 | 0.767 | 0.383 |
| 7 (172) | 7.962-0.237B | 0.226 | 9.077 | 0.003 |
| 8 (172) | 5.874+0.0349B | 0.0393 | 0.263 | 0.609 |
| 9 (171) | 8.935-0.356B | 0.343 | 22.34 | <0.001 |
| 10 (171) | 6.167-0.0245B | 0.0251 | 0.107 | 0.744 |
| **Month** | **FBG-weight-light, Formula** | **R** | **F** | **P-value** |
| 0 (186) | 2.681+0.102B-0.0002L | 0.201 | 3.854 | 0.023 |
| 1 (186) | 1.659+0.0978B+0.0106L | 0.203 | 3.952 | 0.021 |
| 2 (180) | 2.831+0.0292B+0.00598L | 0.137 | 1.69 | 0.188 |
| 3 (180) | 3.086-0.0168B+0.0253L | 0.369 | 13.962 | <0.001 |
| 4 (177) | 4.4-0.231B+0.0415L | 0.571 | 42.16 | <0.001 |
| 5 (176) | 3.505+0.0901B+0.0422L | 0.382 | 14.737 | <0.001 |
| 6 (172) | 3.852+0.147B+0.0175L | 0.219 | 4.23 | 0.016 |
| 7 (172) | 3.844+0.0082B+0.0465L | 0.494 | 27.09 | <0.001 |
| 8 (172) | 4.09+0.0707B+0.0201L | 0.229 | 4.675 | 0.011 |
| 9 (171) | 5.247-0.137B+0.0417L | 0.528 | 32.279 | <0.001 |
| 10 (171) | 4.809 +0.0558B+0.0154L | 0.16 | 2.212 | 0.113 |
| **Month** | **FBG-age-weight-light, Formula** | **R** | **F** | **P-value** |
| 0 (186) | 1.91+0.0648A+0.0985B-0.0014L | 0.344 | 8.128 | <0.001 |
| 1 (186) | 0.751+0.0763A+0.0932B+0.0091L | 0.367 | 9.418 | <0.001 |
| 2 (180) | 2.101+0.0653A+0.0187B+0.005L | 0.35 | 8.207 | <0.001 |
| 3 (180) | 1.964+0.1A-0.0329B+0.0238L | 0.459 | 15.647 | <0.001 |
| 4 (177) | 3.778+0.0555A-0.24B+0.0408L | 0.583 | 29.62 | <0.001 |
| 5 (176) | 2.871+0.0562A+0.0809B+0.0415L | 0.396 | 10.652 | <0.001 |
| 6 (172) | 3.727+0.0115A+0.145B+0.0147L | 0.221 | 2.858 | 0.039 |
| 7 (172) | 4.598-0.0686A+0.0211B+0.0473L | 0.514 | 19.978 | <0.001 |
| 8 (172) | 3.889+0.0185A+0.0671B+0.0199L | 0.233 | 3.227 | 0.024 |
| 9 (171) | 5.818-0.0526A-0.126B+0.0424L | 0.54 | 22.722 | <0.001 |
| 10 (171) | 4.368+0.0409A+0.0471B+0.0148L | 0.185 | 1.964 | 0.121 |
| G, the predicted FBG concentration derived from factors A (age), B (weight), L (light), and any combination of A, B, and L. | | | | |
